# Perturbation of Organogenesis by the Herbicide Atrazine in the Amphibian *Xenopus laevis*

**DOI:** 10.1289/ehp.10742

**Published:** 2007-11-22

**Authors:** Jenny R. Lenkowski, J. Michael Reed, Lisa Deininger, Kelly A. McLaughlin

**Affiliations:** Department of Biology, Tufts University, Medford, Massachusetts, USA

**Keywords:** atrazine, development, morphogenesis, organogenesis, teratogens, *Xenopus laevis*

## Abstract

**Background:**

Exposure to anthropogenic chemicals during development can disrupt the morphogenesis of organ systems. Use of the herbicide atrazine has been debated in recent years because of its implicated, but poorly characterized, effects on vertebrates. Previous studies primarily examined the effects of atrazine exposure during metamorphosis or early developmental stages of amphibians.

**Objectives:**

We sought to identify and characterize the susceptibility during the often-overlooked developmental stage of organ morphogenesis.

**Methods:**

We used a static renewal experimental treatment to investigate the effects of 10, 25, and 35 mg/L atrazine from early organ morphogenesis through the onset of tadpole feeding in the aquatic amphibian model system, *Xenopus laevis*. We quantified malformations of the body axis, heart, and intestine, as well as apoptosis in the midbrain and pronephric kidney.

**Results:**

We found a significant dose-dependent increase in the percentage of atrazine-exposed tadpoles with malformations of multiple tissues including the main body axis, circulatory system, kidney, and digestive system. Incidence of apoptotic cells also increased in the both midbrain and kidney of atrazine-exposed tadpoles.

**Conclusions:**

Our results demonstrate that acute atrazine exposure (10–35 mg/L for ≤ 48 hr) during early organ morphogenesis disrupts proper organ development in an amphibian model system. The concurrent atrazine-induced apoptosis in the pronephric kidney and midbrain begins to elucidate a mechanism by which atrazine may disrupt developmental processes in nontarget organisms.

Previous studies examining several vertebrates have shown that exposure to pesticides can result in detrimental effects on developing nontarget organisms both in natural habitats and in the laboratory (Gilbert and Bolker 2001; Matsushita et al. 2006; Rohr et al. 2006). In recent years, worldwide amphibian population declines have escalated concerns over the potentially harmful effects of pesticides on these sentinel organisms. Despite evidence that shows specific early developmental stages of the vertebrate life cycle are sensitive to environmental contaminants (Greulich and Pflugmacher 2003; Howe et al. 1998; Loewengart 2001; Owens and Baer 2000), many studies are focused on the potential teratogenicity of synthetic substances at later developmental end points, including reproductive development and metamorphosis, or they use chronic exposure over many distinct developmental stages. However, to determine the teratogenicity of a xenobiotic agent, it is important to examine several developmental processes, at multiple stages, to ensure that important environmental health and developmental consequences of chemical exposure are not overlooked.

Use of the herbicide atrazine (2-chloro-4-ethylamine-6-isopropylamino-S-triazine) has been widely debated because of its implicated effects on nontarget organisms. Atrazine is used to control broadleaf weeds by inhibiting electron transport during photosynthesis (Eisler 1989). Importantly, atrazine formulations may be applied to crops at concentrations as high as 213 g/L according to label instructions (Syngenta Crop Protection Inc. 2005), many times its reported solubility in water (30 mg/L). This can lead to “spikes” in the atrazine concentration of agricultural runoff, causing areas with shallow water tables to be particularly susceptible to contamination (Lloyd-Smith et al. 1999; Thurman et al. 1991). Atrazine concentrations as high as 12.7 mg/L have been detected in natural waters (Bushway et al. 1991) and concentrations ≤ 4.9 mg/L were reported in tail water pits used for agricultural impoundment [Davies et al. 1994; Eisler 1989; Kadoum and Mock 1978; Solomon et al. 1996; U.S. Environmental Protection Agency (EPA) 2002]. Atrazine also has a high leaching potential, as it is moderately hydrophilic with high aqueous solubility (Graymore et al. 2001), and it must be applied to crops multiple times early in the growing season. As a result, considerable concentrations of atrazine and its metabolites in groundwater and surface runoff are of concern (Graymore et al. 2001; Lloyd-Smith et al. 1999). Atrazine is very stable, with a half-life of 30–740 days in laboratory and field environments (Comber 1999; Solomon et al. 1996), and two of its mobile breakdown products, deethylatrazine and deisopropylatrazine, are structurally and toxicologically similar to the parent compound (Liu et al. 1996, 1997; Shipitalo and Owens 2003). Although suggested agricultural practices have begun to address these concerns, actual reported levels of atrazine application on corn fields were unchanged between 1991 and 2005 [U.S. Department of Agriculture (USDA) 1991, 2006].

Atrazine was originally thought to be innocuous to animals (Solomon et al. 1996); however, the combination of atrazine’s stability, mobility, and extensive use contribute to water contamination at a time coinciding with amphibian mating and early development in the field. Consequently, atrazine’s potential influence on health problems related to cancer incidence, reproductive endocrinology, and organ development in nontarget vertebrates are of great concern. Because low background levels of atrazine can be found throughout the period of gonadal development in many animals near agricultural fields, several recent studies have focused on examining the effects of atrazine exposure on these developmental stages of amphibians. Chronic atrazine exposure can lead to an increase of intersex gonadal tissues after chronic exposure (*Rana pipiens* and *Xenopus laevis*) (Carr et al. 2003; Hayes et al. 2002, 2003), and as little as a 48-hr exposure can diminish reproductive capacity in male and female frogs (*X. laevis*) (Tavera-Mendoza et al. 2002a, 2002b). In contrast, similar experiments have shown no treatment-specific effect of atrazine exposure on gonad development (Coady et al. 2005; Hecker et al. 2005) or sex-specific traits (Carr et al. 2003) in *X. laevis*. Although these studies indicate the potential for brief and chronic atrazine exposure to disrupt organ development, gonadal development occurs relatively late in development. Thus, it is also important to examine earlier developmental end points to fully evaluate the potential impact of atrazine exposure on individual and population fitness in amphibians.

Several studies have reported a wide range of atrazine-induced effects on non-reproductive organs and tissues. For example, in amphibians, atrazine can affect plasma thyroxine and corticosterone (75 and 250 μg/L; *Ambystoma tigrinum*) (Larson et al. 1998); endocrine function of adrenal cells *in vitro* (2.16 mg/L; *Rana catesbeiana*) (Goulet and Hontela 2003); rate of metamorphosis (800 μg/L; *X. laevis*) (Freeman et al. 2005; Freeman and Rayburn 2005); larval mass (200 μg/L; *Rana sphenocephala* and *Bufo americanus*) (Boone and James 2003); body condition (20 mg/L; *R. pipiens, Rana sylvatica*, and *B. americanus*) (Allran and Karasov 2001); and survival (3 μg/L; *B. americanus*, *Rana clamitansa*, *R. sylvatica*, and *Pseudacris cruicifer*) (Storrs and Kiesecker 2004). These studies show that a wide range of atrazine concentrations can cause detrimental effects on different amphibian species. However, many factors confound the interpretation of these studies because larvae were wild caught or were already feeding at the start of the experiment. Additionally, atrazine exposure during rat embryogenesis has been shown to delay mammary gland development in females (Rayner et al. 2005) and suppress the immune response of males (Rooney et al. 2003). Together, these studies demonstrate that amphibians and mammals exhibit numerous other atrazine-induced effects in addition to those reported for reproductive organ development.

Unlike previous research that examined the effects of atrazine exposure on amphibians and focused on either extremely early (Allran and Karasov 2001; Howe et al. 1998; Morgan et al. 1996) or very late (Carr et al. 2003; Hayes et al. 2003) developmental time points, our analyses focused on evaluating the effects of atrazine exposure on the intervening period of organ morphogenesis using the *X. laevis* amphibian model system. To address the diverse methods used in previously reported studies, we examined the effects of different vehicles used to administer atrazine on the survival and frequencies of observed malformations. We then systematically examined the effects of atrazine exposure during organ morphogenesis (stages 40–47) (Nieuwkoop and Faber 1994), stages that we found to be particularly sensitive to atrazine exposure in preliminary experiments. We evaluated resultant malformations of the intestine, circulatory system, and body axis, and we examined levels of apoptosis in several tissue types to begin to understand the underlying causes of the aberrant phenotypes. Our goal was to use developmental biology to more specifically describe and explain observed effects of atrazine exposure reported in toxicologic studies. To this end, we have identified a window of development that is extremely sensitive to acute atrazine exposure, and have begun to describe the underlying cause of malformations.

## Materials and Methods

### Animal husbandry and embryo manipulation

Adult *Xenopus laevis* (Nasco, Fort Atkinson, WI), were housed under a 14 hr:10 hr light:dark cycle at 18° ± 1°C. Adult females were injected in the dorsal lymph sac with chorionic gonadotropin to induce ovulation. Eggs were collected, fertilized *in vitro* (Peng 1991), dejellied with 2% l-cysteine (Thermo Fisher Scientific Inc., Waltham, MA), and reared at 14–18°C in 0.1X Marc’s Modified Ringer’s Solution (MMR; 1 M NaCl, 20 mM KCl, 10 mM MgCl_2_, 20 mM CaCl_2_, 50 mM HEPES, pH 7.5; Thermo Fisher Scientific Inc.) until they reached the appropriate experimental stage. All embryos were staged according to Nieuwkoop and Faber (NF) (1994). All animals were treated humanely and with regard for alleviation of suffering according to approved protocols (Institutional Animal Care and Use Committee protocol M2005–01).

### Chemical doses and exposures

All reagents were obtained from Thermo Fisher Scientific Inc. unless otherwise noted. We prepared a stock solution containing 5 or 10 g/L atrazine (98% pure; Chem Service, West Chester, PA) in ethanol (EtOH) or dimethyl sulfoxide (DMSO), respectively; stock solutions were prepared fresh monthly or as needed. All treatments consisted of the specified stock diluted to the desired concentration in 0.1X MMR, prepared fresh weekly or as needed. We used three nominal atrazine doses that we identified as producing overt phenotypes: 10, 25, and 35 mg/L. All exposures were performed two or three times with different tadpole broods.

Our preliminary experiments showed the window of development from NF stages 40–47 (organ morphogenesis) to be particularly sensitive to atrazine-induced malformations. This period of development is just before the onset of feeding, and is designated as “premetamorphosis” (includes NF stages 45–53). All experiments were concluded by NF stage 47, because all acute exposures we performed past this stage resulted in 100% mortality (data not shown). All experiments included age-matched, untreated, and vehicle-treated tadpole siblings to ensure that any teratogenic effects observed after atrazine exposure were not the result of parental effects or the vehicle used to prepare the stock solution. Embryos were assigned at random to treatment groups at a density of ≤ 15 tadpoles in 10 mL to prevent overcrowding. Experimental exposures were maintained at 18°C, a temperature common to developmental studies that use *X. laevis*. Experiments were statically renewed every 24 hr and monitored for mortality and tissue malformations.

To ensure that the observed phenotypes were specific to atrazine and not to general toxic effects, tadpoles were exposed to one of two other common herbicides that have chemical structures different from atrazine: 30 mg/L glyphosate (Sigma Chemical Co., St. Louis, MO), or 65, 130, or 260 mg/L 2,4-dichloro-phenoxyacetic acid (2,4-D; Sigma Chemical Co.). Stocks of glyphosate and 2,4-D were made in distilled water and diluted to the appropriate concentration in 0.1X MMR. Glyphosate and 2,4-D concentration ranges used were based on tests of glyphosate formulations (Perkins et al. 2000) and the reported EC_50_ (half maximal effective concentration) and LC_50_ (concentration lethal to 50%) for 2,4-D (245 and 254 mg/L, respectively) (Morgan et al. 1996).

### Phenotype analysis

At the established experimental end point, tadpoles were anesthetized with 0.1% tricaine (ethyl 3-amino-benzoate methanesulfonate salt; Sigma Chemical Co.), scored for circulatory malformations (visible hemorrhaging), and humanely euthanized. Embryos and tadpoles were then fixed in MEMFA (0.1 M MOPS, pH 7.5; 2 mM EGTA; 1 mM MgSO_4_; 3.7% formaldehyde) for 1 hr, rinsed in phosphate-buffered saline (PBS), dehydrated, and stored in methanol. Tadpoles were objectively scored for edema and malformations of the heart, intestine, and axis. Phenotypes were analyzed using standard microscopy and documented using a Nikon SMZ-1500 Stereo Microscope (Nikon Instruments Inc., Melville, NY) with SpotInsight Color Digital Camera (Diagnostik Instruments, Inc., Sterling Heights, MI). Mortality was < 5% in experimental treatments and did not differ among treatment groups. Differences in sample sizes were caused by variations between replicate experiments.

### Immunohistochemistry

Fixed tadpoles were rehydrated in PBTr (1X PBS, 2 mg/mL bovine serum albumin, 0.1% Triton X-100) and incubated with primary antibody overnight at 4°C. After several PBTr washes, tadpoles were incubated overnight with anti-mouse alkaline phosphatase-conjugated (1:1500) or anti-rabbit Alexa Fluor 555-conjugated secondary antibody (1:300; Invitrogen, Eugene, OR). Antibody localization was then visualized with a chromogenic reaction using BCIP (5-bromo-4-chloro-3′-indolyphosphate *p*-toluidine salt; Roche, Indianapolis, IN) and NBT (nitro-blue tetra-zolium chloride; Roche) or standard fluorescent microscopy. Tadpole hearts were visualized using a heart-specific antibody, anti-cardiac troponin-T (CT-3; Developmental Studies Hybridoma Bank, University of Iowa, Iowa City, IA); apoptotic cells were visualized using an antibody to activated caspase-3 (1:250; BD PharMingen, San Diego, CA) (Das et al. 2002; Tseng et al. 2007). Caspase-3 was quantified in the midbrain by counting all positive cells in the midbrain between the eyes and pronephric kidney. Apoptosis in pronephric kidneys was scored on a scale of 0 to 5: 0, no increased apoptosis observed in either kidney; 1, one kidney with faint increase or a few apoptotic cells; 2, both kidneys with faint increase or a few apoptotic cells; 3, one kidney with overt apoptosis throughout the organ; 4, one kidney with overt increase and the contralateral kidney with faint increase or a few apoptotic cells; and 5, both kidneys with overt apoptosis throughout. Scores for each treatment at each time point were averaged and analyzed.

### Data analysis

We used logistic regression to analyze the effects of treatment on the incidence of intestinal malformations, cardiac malformations, axis malformations, edema, and hemorrhaging in tadpoles exposed from NF stage 41 through NF stage 46. Coincidence of phenotypes in individual tadpoles was not analyzed because data on individuals were not kept. We evaluated the effects of treatment, stage at the beginning of exposure (start stage) and interactions between start stage and treatment. To increase statistical power in post hoc tests of treatment effects, we analyzed *a priori* pair-wise contrasts between atrazine treatments and matched vehicle controls. Apoptosis in the midbrain was analyzed with mixed model analysis of variance where replicates were treated as a random effect. The effects of atrazine on midbrain apoptosis was analyzed by time point, and post hoc tests were performed on specified contrasts. Because apoptosis in the pronephric kidney was scored as an ordinal variable, data were analyzed using ordinal regression with the same *a priori* contrasts comparing treatment effects. All analyses were performed with SAS 9.1 (SAS Institute Inc., Cary, NC).

## Results

### Assessment of vehicle

We compared the incidence of atrazine-induced phenotypes using either EtOH or DMSO as the vehicle during a 48-hr exposure (NF stages 42–46; Table 1). DMSO vehicle controls showed a much lower incidence of malformations than EtOH controls, whereas the incidence of phenotypes in atrazine-exposed tadpoles were similar using stock solution made with either DMSO or EtOH vehicle. Therefore, we performed subsequent quantification of atrazine-induced phenotypes using the DMSO vehicle.

### Characterization of organ and tissue malformations

#### Heart and visceral hemorrhaging

**Figure 1:**
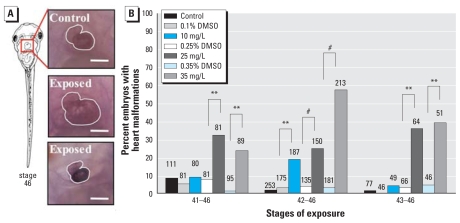
Incidence of heart malformations. (*A*) Ventral views of normal NF stage 46 heart morphology (top) in an untreated-control tadpole, and hypertrophic (middle) and reduced (bottom) hearts in age-matched tadpoles exposed to 35 mg/L atrazine (DMSO) from NF stage 41–46. Hearts were visualized using the CT-3 antibody and are outlined in white. Bars = 0.2 mm. (*B*) Incidence of reduced and enlarged hearts in tadpoles exposed to 10, 25, or 35 mg/L atrazine or vehicle (DMSO) from NF stage 41, 42, or 43 through NF stage 46. Sample sizes are indicated above bars. **p < 0.001, and ^#^*p* < 0.0001, as determined by post hoc pair-wise contrasts from logistic regression.

**Figure 2:**
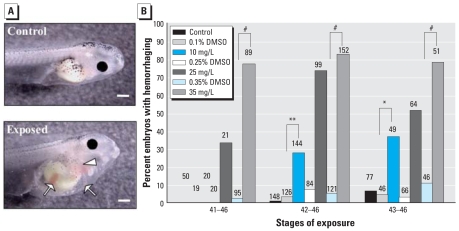
Incidence of visceral hemorrhaging. (*A*) Lateral views, anterior facing right, of a control tadpole (top) and the visceral hemorrhaging phenotype observed near the opercular fold (arrowhead) and in the gut region (arrows) of a tadpole exposed to 35 mg/L atrazine (DMSO) (bottom) from NF stages 42–46. Bars = 0.5 mm. (*B*) Incidence of visceral hemorrhaging in tadpoles exposed to 10, 25, or 35 mg/L atrazine or vehicle (DMSO) from NF stage 41, 42, or 43 through NF stage 46. Sample sizes are indicated above bars. *p < 0.05, **p < 0.001, and ^#^*p* < 0.0001 as determined by post hoc pair-wise contrasts from logistic regression.

Two of the most obvious phenotypes that we observed in tadpoles exposed to atrazine during organ morphogenesis were malformed hearts and visceral hemorrhaging (Figures 1–2). Atrazine exposure significantly increased heart malformations across all treatments [Wald χ^2^ = 162.3, degrees of freedom (df) = 6, *p* < 0.0001, pseudo-*r*^2^ = 0.34]. We observed heart anomalies as early as 24 hr after the initiation of chemical exposure (36% and 39% of tadpoles treated with 25 and 35 mg/L atrazine, respectively; NF stages 43–46). We also observed a significant interaction between treatment and exposure start stage (Wald χ^2^ = 47.8, df = 12 *p* < 0.0001). After 48 hr of exposure (NF stages 42–46), atrazine-treated tadpoles exhibited significantly more abnormal hearts (up to 57% when exposed to 35 mg/L atrazine) than did vehicle controls (3%, 0.35% DMSO control) (Figure 1B). Heart malformations were characterized mainly by an overt reduction in size compared with stage-matched control siblings, although a small number of atrazine-exposed tadpoles exhibited enlarged, hypertrophic cardiac tissue (Figure 1A). Although the general shape of the heart was severely distorted, CT-3 protein was detected throughout cardiac muscle tissue in all tadpoles examined.

In addition to abnormal heart development in atrazine-treated tadpoles, we observed a significant increase in visceral hemorrhaging due to atrazine treatment, visualized as blood pooling (Wald χ^2^ = 150.4, df = 6, *p* < 0.0001, pseudo-*r*^2^ = 0.60). Atrazine-treated tadpoles contained numerous atypical pools of blood in several areas, including kidney (pronephros), tail, abdominal cavity, and the area immediately surrounding the heart (Figure 2A). Visceral hemorrhaging could be observed readily in tadpoles exposed to 35 mg/L atrazine after only 6 hr (data not shown), and there was a significant interaction of exposure start stage and treatment (Wald χ^2^ = 21.4, df = 12, *p* = 0.045). After 24 hr of exposure (NF stages 44–46), this blood pooling phenotype was observed in 79% of tadpoles exposed to 35 mg/L atrazine. In fact, blood pooling occurred over the greatest number of stages, at both 10 and 35 mg/L atrazine (Figure 2B) and was visible after only short windows of exposure (6 hr; data not shown).

#### Intestinal coiling

**Figure 3:**
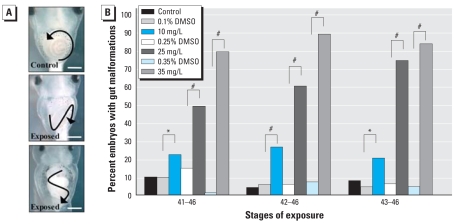
Incidence of gut malformations. (*A*) Ventral views of normal counter-clockwise intestinal coiling of an NF stage 46 untreated control tadpole (top), and mild (middle) and severe (bottom) gut coiling malformations observed in sibling tadpoles exposed to 35 mg/L atrazine (DMSO) from NF stages 42 to 46. Arrows reflect direction of coiling; bars = 0.5 mm. (*B*) Incidence of gut coiling malformations observed in tadpoles exposed to 10, 25, or 35 mg/L atrazine or vehicle (DMSO) from NF stage 41, 42, or 43 through NF stage 46. Sample sizes are same as in Figure 1. *p < 0.05, and ^#^*p* < 0.0001 as determined by post hoc pair-wise contrasts from logistic regression.

Compared with vehicle controls, tadpoles exposed to atrazine exhibited a significant increase in intestine coiling malformations in the form of reduced or aberrant intestinal rotation (Figure 3A; NF stages 41, 42, and 43; all *p* < 0.05). Intestinal miscoiling was observed in > 20%, > 50%, and > 75% of tadpoles exposed to 10, 25, and 35 mg/L atrazine, respectively, during all experimental windows of development examined (Figure 3B). We detected this phenotype after only a short pulse of exposure to atrazine in approximately 80% of tadpoles treated with 35 mg/L atrazine for approximately 36 hr (NF stages 43–46). There was a significant interaction of exposure start stage and treatment (Wald χ^2^ = 32.1, df = 12, *p* < 0.01); within 48 hr of exposure (NF stages 42–46), 90% of the tadpoles exhibited abnormal intestine morphology. Differences in the frequency of this malformation were significant between atrazine-exposed and vehicle control treatments for all windows of development examined (Figure 3B; Wald χ^2^ = 511.5, df = 6, *p* < 0.0001, pseudo-*r*^2^ = 0.54).

#### Body axis and edema

**Figure 4:**
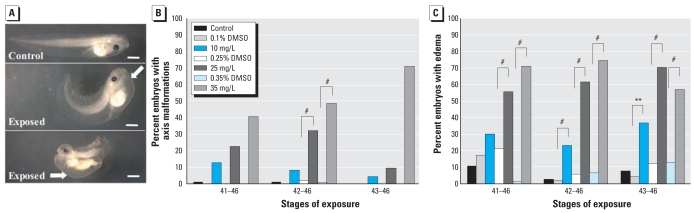
Axis malformations and incidence of edema. (*A*) Lateral view of normal body axis in control NF stage 46 tadpole (top), and atrazine-exposed tadpoles exhibiting curved (middle) and severely shortened (bottom) axes. White arrows indicate edemas observed in the heart (middle) and gut (bottom) regions of atrazine-treated tadpoles. Bars = 1.0 mm. Incidence of axis malformations (*B*) and edemas (*C*) in tadpoles exposed to 10, 25, or 35 mg/L atrazine or vehicle (DMSO) from NF stage 41, 42, or 43 through NF stage 46. Sample sizes for (*B*) and (*C*) are same as in Figure 1. **p < 0.001, and ^#^*p* < 0.0001 as determined by post hoc pair-wise contrasts from logistic regression.

After short periods of exposure (48 hr, NF stages 42–46), atrazine-treated tadpoles exhibited a significant increase of curved body axes relative to stage-matched control or vehicle-treated tadpoles (NF start stages 41, 42, 43; all *p* < 0.0001; Figures 4A, 4B). Affected tadpoles showed tails that curved upward relative to unaffected body (32% and 48% of tadpoles in 25 and 35 mg/L atrazine, NF stages 42–46, respectively). We also found significant effects of treatment (Wald χ^2^ = 110.4, df = 6, *p* < 0.0001, pseudo-*r*^2^ = 0.50) and interactions of start stage and treatment (Wald χ^2^ = 21.2, df = 12, *p* = 0.048) for the incidence of axis malformations. We documented a significant increased incidence of edemas in atrazine-exposed tadpoles (Figure 4A; Wald χ^2^ = 410.4, df = 6, *p* < 0.0001, pseudo-*r*^2^ = 0.45). The frequency of this malformation differed significantly between atrazine-exposed and vehicle-control treatments at nearly all windows of development and treatments examined (Figure 4C; interactions of start stage and treatment, Wald χ^2^ = 46.4, df = 12, *p* < 0.0001).

#### Apoptosis

**Figure 5:**
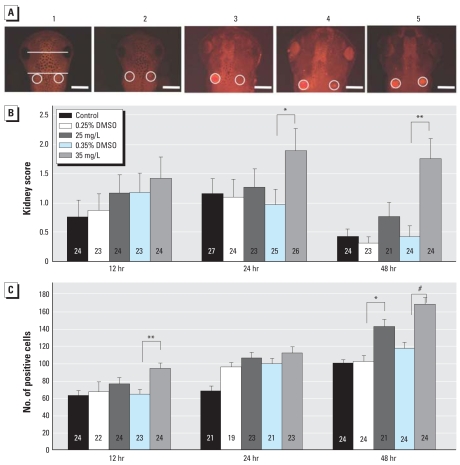
Level of apoptosis in the pronephric kidney and midbrain. (*A*) Dorsal view of tadpoles exhibiting kidney apoptosis phenotypes with corresponding scoring system (0 through 5). Pronephric kidneys are circled in white; the area of midbrain that was analyzed is demarcated in 1 by two white lines. Bars = 0.5 mm. (*B*) Kidney score (mean ± SE) after 12, 24, or 48 hr exposure to 10 or 35 mg/L atrazine or vehicle; data were analyzed using ordinal regression. (*C*) Number of apoptotic cells (mean ± SE) counted in the midbrain between the eyes and pronephric kidney after 12, 24, or 48 hr exposure to 10 or 35 mg/L atrazine or vehicle. *p < 0.05, **p < 0.001, and ^#^*p* < 0.0001 as determined by post hoc pair-wise contrasts.

Preliminary experiments using TdT-mediated 2′-deoxyuridine 5′-triphosphate–nick end labeling (TUNEL; data not shown) indicated increased apoptosis in tadpoles exposed to atrazine. To characterize this effect, we examined immunolocalization of active caspase 3 in the pronephric kidney and midbrain at 6, 12, 24, and 48 hr of exposure, rather than over a range of stages, to clearly determine the timing of this atrazine-induced event. Apoptosis in the kidney was ranked on a scale of 0 to 5, examples of which are shown in Figure 5A, and cells positive for active caspase 3 in the midbrain between the eye and kidney were counted. Statistically similar results were obtained at 6 hr for both apoptosis in the kidney and midbrain across all treatments (data not shown). Tadpoles exposed to 35 mg/L atrazine had significantly more active caspase 3, and thus apoptosis, in the kidneys than the paired DMSO control after 24 hr (Wald χ^2^ = 5.78, *p* = 0.016) and 48 hr of exposure (Wald χ^2^ = 14.73, *p* = 0.0001; (Figure 5B). We observed a significant effect of treatment across all treatment groups after 48 hr of exposure (Wald χ^2^ = 23.16, df = 4, *p* = 0.0001). We also found a significant effect of treatment on the amount of apoptosis in the midbrain after atrazine exposure for 12 hr (*F* = 5.53, df = 4, *p* = 0.0004), 24 hr (*F* = 7.00, df = 4, *p* < 0.0001), and 48 hr (*F* = 12.88, df = 4, *p* < 0.0001) (Figure 5C). After 12 and 48 hr, tadpoles exposed to 35 mg/L atrazine had significantly more apoptosis in the midbrain (*F* = 13.66, *p* = 0.0003 and *F* = 20.33, *p* < 0.0001, respectively); after 48 hr, tadpoles exposed to 10 mg/L atrazine also had significantly more apoptosis in the midbrain (*F* = 11.20, *p* = 0.0011).

#### Assessment of phenotype specificity

In tadpoles exposed to glyphosate (pure compound) from NF stages 42–46, we did not find increased organ malformations compared with sibling-matched controls (data not shown); this is in contrast with our observations in atrazine-exposed tadpoles. Also in contrast, exposure to 2,4-D from stages 42–46 resulted in 100% mortality within 6 hr at all doses tested (65, 130, and 260 mg/L) (data not shown).

## Discussion

Many xenobiotic chemicals are particularly dangerous if present during early animal development because formation of functional tissues and organs is carefully regulated during that time (Broeg et al. 2005; Rayner et al. 2005; Rooney et al. 2003; Wiegand et al. 2001). The present study demonstrates that the developing *X. laevis* tadpole exhibits experimentally significant stage-specific sensitivity to atrazine exposure. In particular, acute exposure to atrazine during the developmental window of early organ morphogenesis (NF stages 40–47), just before the onset of feeding, significantly impacts the formation of multiple organ systems. Interestingly, this window of development coincides with an often-overlooked period of amphibian embryonic development that precedes premetamorphosis. This unexpected result warranted further inquiry, especially because exposure during such a narrow developmental window resulted in a specific set of teratogenic effects on multiple organs and tissues after only a short duration of atrazine exposure.

Because atrazine exposure disrupts normal endocrine function, the majority of previous studies focused on later periods of development, specifically during metamorphosis and gonadal development (Allran and Karasov 2001; Carr et al. 2003; Hayes et al. 2003; Tavera-Mendoza et al. 2002a, 2002b). Because of conflicting reports about the effects or lack of effects that atrazine exposure has during this developmental period, there has been considerable controversy both within the scientific community and the public (reviewed by Hayes 2004). Although reproduction and metamorphosis are critical times in an amphibian life cycle, focusing on developmental anomalies during these relatively late stages overlooks earlier and equally detrimental abnormalities that may occur (Rohr et al. 2004; Storrs and Kiesecker 2004). Studies of atrazine’s effects on amphibians during early development have reported high mortality rates over a large range of concentrations (Howe et al. 1998; Morgan et al. 1996; Storrs and Kiesecker 2004), but in the literature there is little investigation of the causes of this mortality. Consistent with previous studies of atrazine exposure during earlier developmental stages (Allran and Karasov 2001; Morgan et al. 1996; Rohr et al. 2004), here we demonstrated the importance of characterizing early manifestations of disrupted development. Doing so can provide earlier developmental end points at which to evaluate effects of contaminant exposure and help to identify mechanisms of contaminant toxicity to further explore. In addition, lethal effects on early life history traits can remove the most susceptible individuals from a population before gonadal development or metamorphosis occur, and might be a more sensitive bioindicator of ecosystem health.

Two assays that use *X. laevis*—the frog embryo teratogenesis assay-*Xenopus* (FETAX) (Fort et al. 2004; Morgan et al. 1996) and the *Xenopus* metamorphosis assay (XEMA) (Opitz et al. 2005)—provide important standardized techniques and information when evaluating anthropologic substances. However, they do not isolate early organ morphogenesis as part of their exposure parameters and therefore overlook effects particular to these essential developmental stages that we found to be sensitive to atrazine exposure. FETAX, which is used to evaluate interruption of critical cellular behaviors during embryogenesis, relies on a 96-hr exposure protocol beginning early in development through all major early developmental processes such as gastrulation, neurulation, and organogenesis (NF stages 8–46) and analyzes severe end points such as lethality [American Society for Testing and Materials (ASTM) 1988]. Although useful in many respects, examination of such end points after chronic exposure is not sensitive enough to identify the brief developmental window we found to be particularly sensitive to acute atrazine exposure and may not provide clues as to the mechanism of toxicity. In fact, we found that initiating an acute atrazine exposure at the end of the 96-hr FETAX protocol (~ NF < stage 44) and just before the start of the XEMA assay stages (NF stage 52 and later) resulted in severely abnormal organ phenotypes and increased apoptosis (Figures 1–5). Importantly, these sublethal phenotypes were observed in many different organs and tissue types, thereby indicating that atrazine may be perturbing general mechanisms of tissue modeling during the stages we tested. Although the XEMA assay has not been used to test effects of atrazine in particular, the FETAX assay performed by Morgan et al. (1996) used an atrazine formulation at concentrations of 2–98.0 mg/L (3-fold higher than the highest concentration we present here) and reported an EC_50_ of 33 mg/L and an LC_50_ of 100 mg/L. We found more pronounced effects at lower concentrations with the pure compound (10 mg/L atrazine), indicating that the effects of atrazine have yet to be fully explained. The concentrations and DMSO vehicle we used are similar to those used in the FETAX assay by Fort et al. (2004), although our exposures were over a much narrower window of development and focused on quantifying perturbations of normal organ morphogenesis, thereby extending the findings of their work.

Although a few studies have examined the effects of atrazine exposure during early development in aquatic species (Allran and Karasov 2001; Carr et al. 2003; Storrs and Kiesecker 2004), these studies primarily focused on chronic, low-dose exposure and did not examine the effects of acute exposure. In these studies, abnormal swimming was weakly correlated with larval exposure to atrazine concentrations as low as 25 μg/L (Carr et al. 2003) and edema and abnormal tail flexure at 8 mg/L (Morgan et al. 1996). Because aquatic species in the field are exposed to spikes in the environmental concentrations of herbicides for short periods of time immediately after application (Thurman et al. 1991), it is important to include this parameter when profiling teratogens. We observed developmental anomalies after acute atrazine exposure that mimic conditions of agricultural runoff events following atrazine application in the field. We also observed that several malformations occurred after only 6–12 hr of atrazine exposure. Greulich and Pflugmacher (2003) found the analogous stages of tadpole development in the amphibian *Rana arvalis* to be particularly sensitive to pesticide exposure when early developmental stages were examined as well. Thus, our data demonstrate that multiple organs and tissues are highly sensitive to acute atrazine exposure in a dose-dependent manner during early premetamorphic tadpole stages in a model amphibian species with parallels to native species.

The developmental stages just before metamorphosis are important for many reasons. In tadpoles that are no longer able to rely on the energy stores of the embryonic yolk, tissues, such as those in the head and intestines, undergo rapid morphogenesis to prepare for the onset of feeding. Many factors contribute to this intricate process of organ morphogenesis that we observed to be disrupted after atrazine exposure. During normal gut morphogenesis, well-orchestrated gene expression is required in the developing visceral mesoderm and gut endoderm to initiate morphogenic movements (Tseng et al. 2004); regulation of asymmetric cell proliferation and gene expression (Muller et al. 2003) contribute to the proper coiling of the heart and intestine; and morphology of these developing organs can be confirmed by characteristic gene expression of numerous transcription factors and downstream genes (Chalmers and Slack 1998, 2000). During these crucial stages of morphogenesis, we found that tadpoles treated with atrazine for a short period of time (24–48 hr) displayed severely impaired heart (> 55%) and gut morphogenesis (> 75%) of treated tadpoles, compared with < 10% of control tadpoles (Figures 1 and 3). The frequency of malformations was dose dependent, with the highest doses resulting in 90% of the atrazine-treated tadpoles having an aberrant gut phenotype (Figure 3B). Visceral hemorrhaging was a prominent phenotype that was observed after only 6 hr of exposure. Nearly 100% of atrazine-treated tadpoles contained areas of hemorrhaging at the end of our experiments (Figure 2B). This intriguing phenotype suggests a rapid and detrimental morphogenic consequence of atrazine exposure with many possible causes that will warrant further investigation.

We also observed abnormal tail flexure and shortened body axis in atrazine-treated tadpoles (Figure 4A, B). It is possible that these axis deformities are the underlying cause for previously described swimming abnormalities observed in atrazine-treated tadpoles (Allran and Karasov 2001). Atrazine-treated tadpoles also exhibited the highest incidence of edemas when treatments started at NF stages 41 or 42 (Figure 4C). Edemas were observed in several different regions of the tadpole including head, thoracic cavity (especially surrounding the heart), lateral trunk region, and abdomen (Figures 3A and 4A). It is possible that observed edemas may be a secondary consequence of poor water balance in treated tadpoles containing malformed hearts (Figure 2A) and increased apoptosis in pronephric kidneys (Figure 5A, B), rather than being a direct result of atrazine treatment. This is consistent with reduced heart function and circulatory system failure that has been reported in zebrafish exposed to a range of doses of atrazine at analogous stages (Ton et al. 2006; Wiegand et al. 2001).

The malformations we observed were specifically due to exposure to the chemical atrazine, as two other common herbicides that were tested showed no effect (glyphosate) or 100% mortality after a short exposure (2,4-D). Our results for glyphosate are consistent with published studies that indicate that pure glyphosate has no effect on amphibians, whereas its chemical formula, RoundUp (Monsanto Company, Streetsville, Ontario, Canada), is toxic (Perkins et al. 2000). This may also be true for atrazine (Clements et al. 1997; Morgan et al. 1996); however, our results show that even the pure chemical atrazine can cause detrimental effects, and our results may therefore underestimate the potential impact of atrazine formulations in our experiments. 2,4-D has been reported to be neurotoxic to zebrafish at concentrations similar to those at which we found 100% lethality (Ton et al. 2006), and causes 50% lethality at 254 mg/L *X. laevis*, a much higher concentration than we found to cause 100% lethality (Morgan et al. 1996). Our results also are consistent with previous work that showed atrazine has higher teratogenicity than 2,4-D (Morgan et al. 1996). Furthermore, the atrazine-induced malformations we observed in our experiments do not occur in tadpoles exposed to glyphosate or 2,4-D, indicating they are specific to the chemical atrazine and not merely due to toxicity in the presence of a contaminant.

To investigate possible molecular mechanisms by which atrazine causes malformations in *X. laevis* tadpoles during organ morphogenesis, we examined apoptosis by assessing levels of active caspase 3 protein. Atrazine exposure has been shown to increase apoptosis in preimplantation mouse embryos, as well as decrease development to the blastocyst stage (Greenlee et al. 2004). Separate groups have also shown that atrazine exposure decreases proliferation in human fibroblasts (Manske et al. 2004) and increases apoptosis in cultured human lymphocytes (Roloff et al. 1992) and carp cells (Liu et al. 2006), further supporting the potential for atrazine to cause ectopic programmed cell death. Although apoptosis is a normal cellular process in organ morphogenesis (Coles et al. 1993; Kollros and Thiesse 1985; Pexieder 1975), we uncovered a significant increase of apoptosis in the midbrain and pronephric kidney over controls. Ton et al. (2006) found increased apoptosis only in the brain of zebrafish at the highest atrazine concentration tested, approximately three times our highest concentration. Although they ultimately determined that atrazine is primarily teratogenic in zebrafish rather than neurotoxic, further work investigating potential neurotoxicity of atrazine is warranted in amphibians due to the significant increase of apoptosis we observed in the midbrain.

The effects of environmental contaminants on kidney development depend on the stage of development and functional capacity of the kidney at that stage (Solhaug et al. 2004). Our results indicate that 24–48 hr of exposure to the pure compound atrazine is detrimental to the pronephric kidney. It is possible that this toxicity is due to the filtration of the chemical atrazine or to physiologic stress caused by poor heart function and circulation. The apoptosis we observed in the kidneys of exposed tadpoles may be the primary cause of observed edemas, as poor kidney function would disrupt normal water balance. Rainbow trout renal tubule cells show cytologic changes that would indicate atrazine-induced renal damage, including irregular nuclear outline and heterochromatin condensation following chronic atrazine exposure (Oulmi et al. 1995). Additionally atrazine impairs renal function (Santa Maria et al. 1986) and causes DNA fragmentation (Pino et al. 1988) in rats, suggesting a similar means of atrazine excretion and renal damage in mammals and amphibian tadpoles. The mechanism by which atrazine is toxic to the kidney of exposed tadpoles is currently unknown.

## Conclusions

Although concentrations of atrazine used for our focal experiments were high (10–35 mg/L) relative to background levels measured in the field, they were for short durations (< 2 days) and at the limit of reported water solubility for atrazine. We feel that our exposure parameters are relevant for the purpose of this study to identify atrazine-induced malformations, given that atrazine may be applied to crops at concentrations as high as 213 g/L (Syngenta Crop Protection 2005) and reports describing atrazine concentrations peaking in surface water during early runoff events in spring (Thurman et al. 1991) when tadpoles are developing. The present study then provides a basis for future work aimed at elucidating the underlying molecular mechanism by which atrazine exposure simultaneously impacts so many different organ systems during a finite developmental window by identifying affected organ systems and overt phenotypes. For example, it is not known whether the effects of atrazine on the multiple tissues and organs are interrelated. The increased levels of apoptosis in the midbrain and pronephric kidney we observe suggest that atrazine may cause tissue malformations by inducing ectopic programmed cell death, either directly or indirectly through a mechanism that has not been identified. Ultimately, the identification of sublethal indicators of organism health and environmental contamination provide researchers a sensitive bioassay for monitoring effects of anthropologic agents found in our ecosystems.

## Figures and Tables

**Table 1:** Effect of using different vehicles to deliver atrazine treatments on the incidence of tissue malformations, exposure stages NF 42–46.

	No.		Percent survival		Percent abnormal gut coiling		Percent abnormal hearts		Percent edemas	
Treatment	EtOH	DMSO	EtOH	DMSO	EtOH	DMSO	EtOH	DMSO	EtOH	DMSO
Untreated control	126	253	99	100	4	4	7	2	0	3
25 mg/L atrazine	67	150	93	100	47	61	21	25	21	61
Vehicle control #1	67	135	97	100	17	6	17	4	2	6
50 mg/L atrazine	84	72	95	100	100	100	63	96	93	100
Vehicle control #2	85	69	100	100	12	9	6	3	4	3

## References

[b1-ehp0116-000223] Allran J, Karasov W (2001). Effects of atrazine on embryos, larvae, and adults of anuran amphibians. Environ Toxicol Chem.

[b2-ehp0116-000223] ASTM (1988). Standard guide for conducting the frog embryo teratogenesis assay - *Xenopus*. Designation E 1439–1491. Annual Book of ASTM Standards.

[b3-ehp0116-000223] Boone MD, James SM (2003). Interactions of an insecticide, herbicide, and natural stressors in amphibian community mesocosms. Ecol Appl.

[b4-ehp0116-000223] Broeg K, Westernhagen HV, Zander S, Korting W, Koehler A (2005). The “bioeffect assessment index” (BAI). A concept for the quantification of effects of marine pollution by an integrated biomarker approach. Mar Pollut Bull.

[b5-ehp0116-000223] Bushway RJ, Perkins LB, Fukal L, Harrison RO, Ferguson BS (1991). Comparison of enzyme-linked immunosorbent assay and high-performance liquid chromatography for the analysis of atrazine in water from Czechoslovakia. Arch Environ Contam Toxicol.

[b6-ehp0116-000223] Carr JA, Gentles A, Smith EE, Goleman WL, Urquidi LJ, Thuett K (2003). Response of larval *Xenopus laevis* to atrazine: assessment of growth, metamorphosis, and gonadal and laryngeal morphology. Environ Toxicol Chem.

[b7-ehp0116-000223] Chalmers AD, Slack JM (1998). Development of the gut in *Xenopus laevis*. Dev Dyn.

[b8-ehp0116-000223] Chalmers AD, Slack JM (2000). The *Xenopus* tadpole gut: fate maps and morphogenetic movements. Development.

[b9-ehp0116-000223] Clements C, Ralph S, Petras M (1997). Genotoxicity of select herbicides in *Rana catesbeiana* tadpoles using the alkaline single-cell gel DNA electrophoresis (comet) assay. Environ Mol Mutagen.

[b10-ehp0116-000223] Coady KK, Murphy MB, Villeneuve DL, Hecker M, Jones PD, Carr JA (2005). Effects of atrazine on metamorphosis, growth, laryngeal and gonadal development, aromatase activity, and sex steroid concentrations in *Xenopus laevis*. Ecotoxicol Environ Saf.

[b11-ehp0116-000223] Coles HS, Burne JF, Raff MC (1993). Large-scale normal cell death in the developing rat kidney and its reduction by epidermal growth factor. Development.

[b12-ehp0116-000223] Comber SD (1999). Abiotic persistence of atrazine and simazine in water. Pestic Sci.

[b13-ehp0116-000223] Das B, Schreiber AM, Huang H, Brown DD (2002). Multiple thyroid hormone-induced muscle growth and death programs during metamorphosis in *Xenopus laevis*. Proc Natl Acad Sci USA.

[b14-ehp0116-000223] Davies PE, Cook LSJ, Barton JL (1994). Triazine herbicide contamination of Tasmanian streams: sources, concentrations and effects on biota. Aust J Mar Freshw Res.

[b15-ehp0116-000223] Eisler R (1989). Atrazine hazards to fish, wildlife, and invertebrates: a synoptic review. Biological Report.

[b16-ehp0116-000223] Fort DJ, Rogers RL, Thomas JH, Buzzard BO, Noll AM, Spaulding CD (2004). Comparative sensitivity of *Xenopus tropicalis* and *Xenopus laevis* as test species for the FETAX model. J Appl Toxicol.

[b17-ehp0116-000223] Freeman JL, Beccue N, Rayburn AL (2005). Differential metamorphosis alters the endocrine response in anuran larvae exposed to T_3_ and atrazine. Aquat Toxicol.

[b18-ehp0116-000223] Freeman JL, Rayburn AL (2005). Developmental impact of atrazine on metamorphing *Xenopus laevis* as revealed by nuclear analysis and morphology. Environ Toxicol Chem.

[b19-ehp0116-000223] Gilbert SF, Bolker JA (2001). Homologies of process and modular elements of embryonic construction. J Exp Zool.

[b20-ehp0116-000223] Goulet BN, Hontela A (2003). Toxicity of cadmium, endosulfan, and atrazine in adrenal steroidogenic cells of two amphibian species, *Xenopus laevis* and *Rana catesbeiana*. Environ Toxicol Chem.

[b21-ehp0116-000223] Graymore M, Stagnitti F, Allinson G (2001). Impacts of atrazine in aquatic ecosystems. Environ Int.

[b22-ehp0116-000223] Greenlee AR, Ellis TM, Berg RL (2004). Low-dose agrochemicals and lawn-care pesticides induce developmental toxicity in murine preimplantation embryos. Environ Health Perspect.

[b23-ehp0116-000223] Greulich K, Pflugmacher S (2003). Differences in susceptibility of various life stages of amphibians to pesticide exposure. Aquat Toxicol.

[b24-ehp0116-000223] Hayes T (2004). There is no denying this: defusing the confusion about atrazine. BioScience.

[b25-ehp0116-000223] Hayes T, Collins A, Lee M, Mendoza M, Noriega N, Stuart AA (2002). Hermaphroditic, demasculinized frogs after exposure to the herbicide atrazine at low ecologically relevant doses. Proc Natl Acad Sci USA.

[b26-ehp0116-000223] Hayes T, Haston K, Tsui M, Hoang A, Haeffele C, Vonk A (2003). Atrazine-induced hermaphroditism at 0.1 ppb in American leopard frogs (*Rana pipiens*): laboratory and field evidence. Environ Health Perspect.

[b27-ehp0116-000223] Hecker M, Kim WJ, Park JW, Murphy MB, Villeneuve D, Coady KK (2005). Plasma concentrations of estradiol and testosterone, gonadal aromatase activity and ultra-structure of the testis in *Xenopus laevis* exposed to estradiol or atrazine. Aquat Toxicol.

[b28-ehp0116-000223] Howe GE, Gillis R, Mowbray RC (1998). Effect of chemical synergy and larval stage on the toxicity of atrazine and alachlor to amphibian larvae. Environ Toxicol Chem.

[b29-ehp0116-000223] Kadoum AM, Mock DE (1978). Herbicide and insecticide residues in tailwater pits: water and pit bottom soil from irrigated corn and sorghum fields. J Agric Food Chem.

[b30-ehp0116-000223] Kollros JJ, Thiesse ML (1985). Growth and death of cells of the mesencephalic fifth nucleus in *Xenopus laevis* larvae. J Comp Neurol.

[b31-ehp0116-000223] Larson DL, McDonald S, Fivizzani AJ, Newton WE, Hamilton SJ (1998). Effects of the herbicide atrazine on *Ambystoma tigrinum* metamorphosis: duration, larval growth, and hormonal response. Physiol Zool.

[b32-ehp0116-000223] Liu S, Lu J-C, Kolpin DW, Meeker WQ (1997). Analysis of environmental data with censored observations. Environ Sci Technol.

[b33-ehp0116-000223] Liu S, Yen ST, Kolpin DW (1996). Pesticides in groundwater: do atrazine metabolites matter?. Water Resour Bull.

[b34-ehp0116-000223] Liu XM, Shao JZ, Xiang LX, Chen XY (2006). Cytotoxic effects and apoptosis induction of atrazine in a grass carp (*Ctenopharyngodon idellus*) cell line. Environ Toxicol.

[b35-ehp0116-000223] Lloyd-Smith J, Allinson G, Stagnitti F, Colville S, Cordell S (1999). The fate of atrazine in forestry soil and groundwater [Abstract]. Geophys Res Abstr.

[b36-ehp0116-000223] Loewengart G (2001). Toxicity of boron to rainbow trout: a weight-of-the-evidence assessment. Environ Toxicol Chem.

[b37-ehp0116-000223] Manske MK, Beltz LA, Dhanwada KR (2004). Low-level atrazine exposure decreases cell proliferation in human fibroblasts. Arch Environ Contam Toxicol.

[b38-ehp0116-000223] Matsushita S, Yamashita J, Iwasawa T, Tomita T, Ikeda M (2006). Effects of *in ovo* exposure to imazalil and atrazine on sexual differentiation in chick gonads. Poult Sci.

[b39-ehp0116-000223] Morgan MK, Scheuerman PR, Bishop CS, Pyles RA (1996). Teratogenic potential of atrazine and 2,4-D using FETAX. J Toxicol Environ Health.

[b40-ehp0116-000223] Muller JK, Prather DR, Nascone-Yoder NM (2003). Left-right asymmetric morphogenesis in the *Xenopus* digestive system. Dev Dyn.

[b41-ehp0116-000223] Nieuwkoop PD, Faber J (1994). Normal Table of *Xenopus laevis*.

[b42-ehp0116-000223] Opitz R, Braunbeck T, Bogi C, Pickford DB, Nentwig G, Oehlmann J (2005). Description and initial evaluation of a *Xenopus* metamorphosis assay for detection of thyroid system-disrupting activities of environmental compounds. Environ Toxicol Chem.

[b43-ehp0116-000223] Oulmi Y, Negele RD, Braunbeck T (1995). Segment specificity of the cytological response in rainbow trout (*Oncorhynchus mykiss*) renal tubules following prolonged exposure to sublethal concentrations of atrazine. Ecotoxicol Environ Saf.

[b44-ehp0116-000223] Owens KD, Baer KN (2000). Modifications of the topical Japanese medaka (*Oryzias latipes*) embryo larval assay for assessing developmental toxicity of pentachlorophenol and *p*,*p*′-dichlorodiphenyltrichloroethane. Ecotoxicol Environ Saf.

[b45-ehp0116-000223] Peng HB (1991). *Xenopus laevis*: practical uses in cell and molecular biology. Solutions and protocols. Methods Cell Biol.

[b46-ehp0116-000223] Perkins PJ, Boermans HJ, Stephenson GR (2000). Toxicity of glyphosate and triclopyr using the frog embryo teratogenesis assay-Xenopus. Environ Toxicol Chem.

[b47-ehp0116-000223] Pexieder T (1975). Cell death in the morphogenesis and teratogenesis of the heart. Adv Anat Embryol Cell Biol.

[b48-ehp0116-000223] Pino A, Maura A, Grillo P (1988). DNA damage in stomach, kidney, liver and lung of rats treated with atrazine. Mutat Res.

[b49-ehp0116-000223] Rayner JL, Enoch RR, Fenton SE (2005). Adverse effects of pre-natal exposure to atrazine during a critical period of mammary gland growth. Toxicol Sci.

[b50-ehp0116-000223] Rohr JR, Elskus AA, Shepherd BS, Crowley PH, McCarthy TM, Niedzwiecki JH (2004). Multiple stressors and salamanders: effects of an herbicide, food limitation, and hydro-period. Ecol Appl.

[b51-ehp0116-000223] Rohr JR, Sager T, Sesterhenn TM, Palmer BD (2006). Exposure, postexposure, and density-mediated effects of atrazine on amphibians: breaking down net effects into their parts. Environ Health Perspect.

[b52-ehp0116-000223] Roloff B, Belluck D, Meisner L (1992). Cytogenetic effects of cyanazine and metolachlor on human lymphocytes exposed in vitro. Mutat Res.

[b53-ehp0116-000223] Rooney AA, Matulka RA, Luebke RW (2003). Developmental atrazine exposure suppresses immune function in male, but not female Sprague-Dawley rats. Toxicol Sci.

[b54-ehp0116-000223] Santa Maria C, Vilas MG, Muriana FG, Relimpio A (1986). Subacute atrazine treatment effects on rat renal functions. Bull Environ Contam Toxicol.

[b55-ehp0116-000223] Shipitalo MJ, Owens LB (2003). Atrazine, deethylatrazine, and deisopropylatrazine in surface runoff from conservation tilled watersheds. Environ Sci Technol.

[b56-ehp0116-000223] Solhaug MJ, Bolger PM, Jose PA (2004). The developing kidney and environmental toxins. Pediatrics.

[b57-ehp0116-000223] Solomon KR, Baker DB, Richards RP, Dixon KR, Klaine SJ, La Poine TW (1996). Ecological risk assessment of atrazine in North American surface waters. Environ Toxicol Chem.

[b58-ehp0116-000223] Storrs SI, Kiesecker JM (2004). Survivorship patterns of larval amphibians exposed to low concentrations of atrazine. Environ Health Perspect.

[b59-ehp0116-000223] Syngenta Crop Protection Inc (2005). Aatrex 4L Product Manual.

[b60-ehp0116-000223] Tavera-Mendoza L, Ruby S, Brousseau P, Fournier M, Cyr D (2002a). Response of the amphibian tadpole (*Xenopus laevis*) to atrazine during sexual differentiation of the testis. Environ Toxicol Chem.

[b61-ehp0116-000223] Tavera-Mendoza L, Ruby S, Brousseau P, Fournier M, Cyr D (2002b). Response of the amphibian tadpole *Xenopus laevis* to atrazine during sexual differentiation of the ovary. Environ Toxicol Chem.

[b62-ehp0116-000223] Thurman EM, Goolsby DA, Meyer MT, Kolpin DW (1991). Herbicides in surface waters of the midwestern states: the effect of spring flush. Environ Sci Technol.

[b63-ehp0116-000223] Ton C, Lin Y, Willett C (2006). Zebrafish as a model for developmental neurotoxicity testing. Birth Defects Res A Clin Mol Teratol.

[b64-ehp0116-000223] Tseng AS, Adams DS, Qiu D, Koustubhan P, Levin M (2007). Apoptosis is required during early stages of tail regeneration in *Xenopus laevis*. Dev Biol.

[b65-ehp0116-000223] Tseng HT, Shah R, Jamrich M (2004). Function and regulation of FoxF1 during Xenopus gut development. Development.

[b66-ehp0116-000223] USDA (1991). Agricultural Chemical Usage 1990 Field Crop Summary.

[b67-ehp0116-000223] USDA (2006). Agricultural Chemical Usage 2005 Field Crops Summary.

[b68-ehp0116-000223] U.S. EPA (2002). Drinking Water Standards and Health Advisories. EPA 822-R-02–038.

[b69-ehp0116-000223] Wiegand C, Krause E, Steinberg C, Pflugmacher S (2001). Toxico-kinetics of atrazine in embryos of the zebrafish (*Danio rerio*). Ecotoxicol Environ Saf.

